# *Sthenelais onca* sp. nov. (Phyllodocida, Sigalionidae) from a sandy beach on the North Pacific coast of Costa Rica

**DOI:** 10.3897/zookeys.1278.176443

**Published:** 2026-04-24

**Authors:** Jeffrey A. Sibaja-Cordero, Waiomi Miranda-García

**Affiliations:** 1 Centro de Investigación en Ciencias del Mar y Limnología (CIMAR), Ciudad de la Investigación, Universidad de Costa Rica, San Pedro, San José 11501-2060, Costa Rica Centro de Investigación en Ciencias del Mar y Limnología (CIMAR), Ciudad de la Investigación, Universidad de Costa Rica San José Costa Rica; 2 Escuela de Biología, Universidad de Costa Rica, San Pedro, San José 11501-2060, Costa Rica Escuela de Biología, Universidad de Costa Rica San José Costa Rica; 3 Museo de Zoología, Centro de Investigación en Biodiversidad y Ecología Tropical (CIBET), Universidad de Costa Rica, San Pedro, San José 11501-2060, Costa Rica Museo de Zoología, Centro de Investigación en Biodiversidad y Ecología Tropical (CIBET), Universidad de Costa Rica San José Costa Rica

**Keywords:** COI, falciger, key, neurochaetae, notched elytra, sandy beach, scale worm, smooth shaft

## Abstract

Errant polychaetes of the family Sigalionidae are active, scale-covered predators inhabiting sandy marine bottoms. Knowledge of this family in tropical America is scarce, with only a few species reported from Costa Rica. In this study, seven specimens of *Sthenelais* were collected from the intertidal zone of Playa Naranjo, Área de Conservación Guanacaste, on the North Pacific coast of Costa Rica. Morphological examinations and Bayesian phylogenetic analyses based on DNA barcoding (COI) were conducted to determine their taxonomic identity. The specimens were recovered within the *Sthenelais* clade, forming a distinct and well-supported subclade that includes *S. limicola* from Europe and representatives from Asia, and that differs from western Pacific congeners. Morphologically, the new species *Sthenelais onca***sp. nov**. differs from other Eastern Tropical Pacific congeners by the absence of serrations on the shafts of neurochaetae throughout the body, stylodes without papillae, and anterior elytra bearing a notch on the supra-interior margin that diminishes posteriorly. The species inhabits saturated sand in the intertidal zone and exhibits a unique elytral coloration pattern reminiscent of a jaguar’s coat. This study describes a new species of *Sthenelais* from sandy beaches of the Pacific coast of Costa Rica and provides an updated identification key for the genus in the region, expanding the known diversity and distribution of Sigalionidae in the Tropical Eastern Pacific.

## Introduction

Errant worms within the order Phyllodocida are distinguished by their eversible pharynx; some families in the order have their dorsal side partially or completely covered by scales (Rouse et al. 2022). Among scale worms, the family Sigalionidae Kinberg, 1856 comprises long-bodied, cosmopolitan, active predators that typically inhabit sandy bottoms (Salazar-Silva and Salazar-Vallejo 2021). Knowledge of this family is scarce in tropical America; Salazar-Vallejo and Londoño-Mesa (2004) listed 40 species, including four questionable species, for the Tropical Eastern Pacific.

In Costa Rica, two species *Fimbriosthenelais hobbsi* Pettibone, 1971, and *Pisionidens indica* (Aiyar & Alikuhni, 1940), have been reported from the Caribbean Sea (Dean 2017; Sibaja-Cordero et al. 2018), and nine species are documented along the Pacific Ocean (Dean 2004). At Isla del Coco, records include *Neopsammolyce spinosa* (Hartman, 1939), *Sthenelanella uniformis* Moore, 1910, and *Thalanessa lewisii* (Berkeley & Berkeley, 1939) (Dean 2009; Sibaja-Cordero et al. 2016). *Sigalion spinosum* Hartman, 1939, *Sthenelais fusca* Johnson, 1897, and *S. uniformis* were found in the subtidal sandy and muddy bottoms of the mainland coast (Dean 2004, 2009). Three species from sandy beaches are also part of these records: *Pelogenia anoculata* (Hartman, 1939) described as *Psammolyce antipoda anoculata*, from the North Pacific coast; *Sthenelais helenae* Kinberg, 1856, from the Central Pacific coast; and *Pisione remota* (Southern, 1914), a small species of the subfamily Pisioninae (Dean 2004; Salazar-Silva and Salazar-Vallejo 2021). Finally, two additional taxa were mentioned by Dean (2004): *Sigalion* sp. and another sigalionid sp. from subtidal sediments of the Gulf of Nicoya.

This study is based on specimens of *Sthenelais* found on a sandy beach of the Costa Rican North Pacific coast. *Sthenelais* is characterized by three antennae, the lateral fused at the parapodium, and the median with small auricles; the ventral cirri and stylodes are smooth, and there is no dorsal cirrus on chaetiger 3; dorsal tubercles on segments 3, 6, 8, then alternate elytra until segment 27; the other segments from 2 to 27 have dorsal cirri; and neuropodial falcigers are present (Pettibone 1971; Fauchald 1977). This genus has 38 valid species (Read and Fauchald 2026), but only nine were mentioned for the Pacific of the Americas. In North America, *Sthenelais berkeleyi* Pettibone, 1971 was described from British Columbia, *Sthenelais tertiaglabra* Moore, 1910 and *Sthenelais verruculosa* Johnson, 1897 from California, *Sthenelais neoleanirae* Hartman, 1939 from Baja California (Salazar-Silva and Salazar-Vallejo 2021). In South America *Sthenelais koepckei* Hartmann-Schröder, 1960 was described from Peru, *Sthenelais blanchardi* Kinberg, 1858 and *Sthenelais caerulea* (Schmarda, 1861) was described from Chile (Hartmann-Schröder 1960; Lancellotti and Vásquez 2000). Finally, the species with broad distributions are *S. fusca*, ranging from California to Peru, and *S. helenae* from Mexico to Chile (Salazar-Silva and Salazar-Vallejo 2021). As previously mentioned, these last two species are currently recorded from Costa Rica.

Since 2015, the BioMar-ACG project has aimed to document marine biodiversity in the Área de Conservación Guanacaste (ACG), Costa Rica by collecting, identifying, cataloging, and publishing findings and creating a DNA barcode reference library (Cortés and Joyce 2020). This is an association between a government agency (ACG) and an academic institution, the University of Costa Rica (UCR), specifically through the Centro de Investigación de Mar y Limnología (CIMAR) and the Museo de Zoología (MZUCR). This project allows for the study of lesser-known groups, such as annelids. The present study provides a description of a new species of *Sthenelais* from ACG, Costa Rica, Central America, in the Tropical Eastern Pacific.

## Material and methods

### Study area

**Figure 1.**
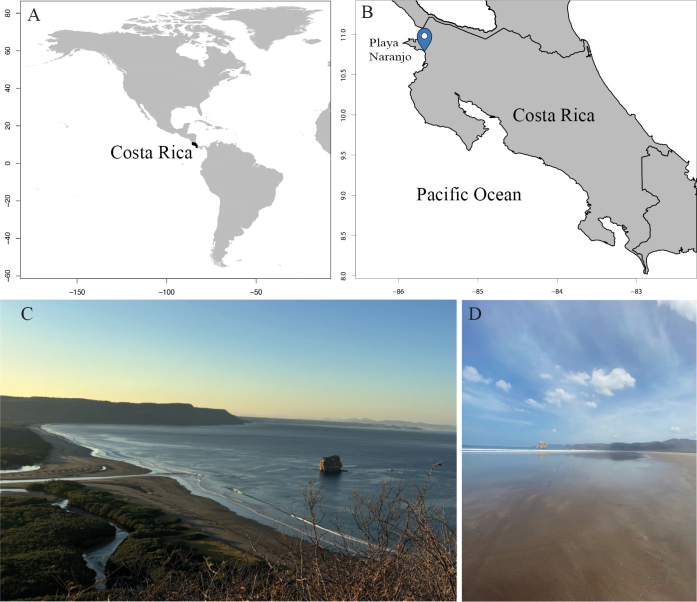
Study site location. **A**. Costa Rica; **B**. Playa Naranjo, Santa Rosa National Park, Guanacaste, Pacific coast; **C**. Sandy beach ecosystem; **D**. Saturated zone of the sandy intertidal.

Naranjo Beach is in Santa Rosa National Park, Guanacaste, Costa Rica (10°47'N, 85°40'W). It is 6 km long and covers an area of 822 533 m^2^ (Fig. 1). Mean annual precipitation is 1623.7 mm yr−1, and mean temperature is 26.7 °C (Calvo-Alvarado et al. 2018). The northern end of the sandy beach, beginning south of Naranjo estuary, is wide with low dunes, while the middle of the beach is still wide, but steeper. A mangrove forest and a lagoon are located behind the beach. The southern end of the beach is narrower and rockier (Hagerty et al. 2003).

Fieldwork to search for polychaetes in the low intertidal and surf zone sand (less than 1 m depth) was conducted in February 2016 and May 2024. Sand was inspected by manually digging several shallow holes (less than 40 cm deep) with shovels, and the material was transferred to a 1 mm sieve to retain polychaetes. Voucher specimens preserved in 95% ethanol were deposited in the Museo de Zoología, Universidad de Costa Rica (**MZ-UCR**, **CIBET**).

### Morphology

To describe the species and compare it with other species of *Sthenelais*, we followed the criteria proposed by Fauchald (1977) and Salazar-Silva and Salazar-Vallejo (2021). A taxonomic review based on morphological characters from the original descriptions of species in the genus *Sthenelais* was conducted to compare and identify morphologically distinctive characters of the proposed species. Seven specimens were dissected and examined using a compound microscope and a stereoscope. Illustrations were executed using stippling and line work to depict morphological structures with precision. A Leica MZ 8 stereomicroscope equipped with a Leica 10308700 drawing tube was used to illustrate ventral and dorsal views of the prostomium and peristomium, middle segments, and pygidium. Digital images of chaetigers 4 and 60, as well as neurochaetae and elytra, were captured with an ICOE BE520T compound microscope at total magnifications of 40× and 1000× via an OMAX A35140U microscope camera operated through ToupView (ToupTek Photonics, 2023, Version 4.11.23274.20230903). The software was employed for image acquisition, adjustment of brightness and contrast, and for obtaining basic linear measurements.

### DNA extraction, amplification, and sequencing

Tissue samples of five specimens of Sigalionidae preserved in 95% ethanol were shipped to the Canadian Centre for DNA Barcoding (CCDB), where the standard COI DNA barcode was sequenced from each specimen using CCDB protocols (Ivanova et al. 2006). PCR products were Sanger sequenced using the primers polyLCO (Forward) 5'– GAYTATWTTCAACAAATCATAAAGATATTGG–3' and polyHCO (Reverse) 5'– TAMACTTCWGGGTGACCAAARAATCA–3' (Carr et al. 2011). Sequences and detailed specimen information were stored in the Barcode of Life Data Systems (BOLD) under the project BMAR: “Specimen from Sector Marino of ACG (BioMar)”. Sequence data were exported from BOLD in FASTA format.

COI sequences of the available species of the subfamilies Sigalioninae and Sthenelanellinae were downloaded from GenBank and BOLD (Table 1). Sequences were aligned using MAFFT (version 7.407_1) (Katoh and Standley 2013). Subsequently, a Bayesian phylogenetic analysis was conducted in MrBayes (version 3.2.7a) (Huelsenbeck and Ronquist 2001). The General Time Reversible model with corrections for a discrete gamma distribution and a proportion of invariant sites (GTR+Γ+I) was applied and partitioned by codon (see Suppl. material 1). This is the evolutionary model with the lowest AIC and BIC values during model selection in MEGA 12 (Kumar et al. 2024). The analysis was conducted with two runs and four Markov chains, using a burn-in fraction of 50%, 1,000,000 generations, and a sampling interval of 100 steps. Convergence was detected in Tracer (version 1.7.2) (Rambaut et al. 2018).

**Table 1.** Species of Sigalionidae included in the COI molecular analysis, country of collection, GenBank accession numbers, BOLD ID code, reference, and voucher depository. Sequences of *Aphrodita aculeata* and *Mexieulepis weberi* represent the outgroups.

Species	Country	GB Accession	BOLD ID	Reference	Voucher Depository
*Pisionidens* sp.	Mexico	JN852943.1	-	Norlinder et al. (2012)	Swedish Museum of Natural History
*Pisionidens ixazaluohae*	Mexico Caribbean	KX282505.1	-	Petersen et al. (2016)	National History Museum of Denmark (ZMUC)
*Pisione papuensis*	Australia	KY657664.1	-	Gonzalez et al. (2017)	-
*Pisione remota*	Sweden	KY657668.1	-	Gonzalez et al. (2017)	-
*Pholoides asperus*	USA West Coast	JN852942.1	-	Gonzalez et al. (2018)	Swedish Museum of Natural History (SMNH), Sweden
*Pholoe pallida*	Great Britain, UK	MN165384.1	-	Meißner et al. (2020)	University Museum of Bergen (ZMBN), Norway
*Pholoe assimilis*	Norway	MN165336.1	-	Meißner et al. (2020)	University Museum of Bergen (ZMBN), Norway
*Laubierpholoe* sp. B	Mexico Caribbean	KY823499.1	-	Gonzalez et al. (2018)	-
*Euthalenessa festiva*	China	KY753837.1	-	Zhang et al. (2018)	-
*Fimbriosthenelais zetlandica*	Spain	KX946850.1	-	Ravara et al. (2017)	-
*Fimbriosthenelais zetlandica*	India	OQ417171.1	-	-	CSIR National Institute of Oceanography
*Labioleanira yhleni*	Spain	KT307649.1	-	Aylagas et al. (2016)	-
*Labiosthenolepis laevis*	New Zealand	-	JBMFE021-22	-	National Institute of Water and Atmospheric Research, Wellington
*Leanira hystricis*	India	OQ417177.1	-	-	-
*Leanira hystricis*	Norway	-	POLNB1763-15	-	University of Bergen, Natural History Collections
*Leanira quatrefagesi*	Chile	GQ229114.1	-	Canales-Aguirre et al. (2011)	-
*Leanira quatrefagesi*	Chile	GQ229115.1	-	Canales-Aguirre et al. (2011)	-
*Leanira quatrefagesi*	Chile	GQ229116.1	-	Canales-Aguirre et al. (2011)	-
*Leanira* sp. 22 PB	Clipperton Pacific Ocean	-	CCFZP348-19	-	Institut Francais de Recherche Pour l’Exploitation de la Mer (IFREMER)
*Leanira* sp. 453b PB	Clipperton Pacific Ocean	-	CCFZP485-19	-	Institut Francais de Recherche Pour l’Exploitation de la Mer (IFREMER)
*Leanira* sp.	USA East Coast	AY894315.1	-	Struck et al. (2005)	-
*Neoleanira tetragona*	Norway	AY839582.1	-	Wiklund et al. (2005)	-
*Neoleanira tetragona*	Norway	-	PONOR089-13	-	NTNU University Museum, Department of Natural History
*Neoleanira tetragona*	Norway	AY839582.1	-	Wiklund et al. (2005)	-
*Sigalion bandaensis*	Australia	AY583699.1	-	Colgan et al. (2006)	-
*Sigalion mathildae*	North Sea	-	GEANS165-20	-	Deutsches Zentrum fuer Marine Biodiversitaets forschung
*Sigalion mathildae*	North Sea	PQ739595.1	-	Christodoulou et al. (2025)	-
*Sigalion mathildae*	Italy	ON716074.1	-	Mugnai et al. (2023)	-
*Sigalion spinosa*	USA West	AY894319.1	-	Struck et al. (2005)	-
*Sigalion spinosus*	USA West	-	CMBIA297-11	-	Natural History Museum of Los Angeles County
*Sigalion squamosus*	Norway	-	POLNB1631-15	-	University of Bergen, Natural History Collections
Sigalionidae gen. sp. 210	Clipperton Pacific Ocean	-	CCFZP312-19	-	Institut Francais de Recherche Pour l’Exploitation de la Mer
*Stenolepis izuensis hwanghaiensis* as *Ehlersileanira izuensis hwanghaiensis*	China	-	HZPLY1012-13	-	Centre for Biodiversity Genomics
*Sthenelais berkeleyi*	West USA	-	BBPS790-19	-	Florida Museum of Natural History
*Sthenelais berkeleyi*	West USA	MH242982.1	-	-	Smithsonian Tropical Research Institute
*Sthenelais boa*	India	KY783698.1	-	Vijapure et al. (2019)	-
*Sthenelais boa*	India	KY775639.1	-	Vijapure et al. (2019)	-
*Sthenelais boa*	France	KJ183006.1	-	Cowart et al. (2015)	-
*Sthenelais boa*	Portugal	KR916939.1	SFPOM068-11	Lobo et al. (2016)	-
*Sthenelais boa*	USA East	KP254706.1	VATWO031-14	Leray and Knowlton (2015)	-
*Sthenelais boa*	USA East	KP254691.1	VATWO027-14	Leray and Knowlton (2015)	-
*Sthenelais boa*	USA East	AY894321.1	-	Struck et al. (2005)	-
*Sthenelais boa*	India	MZ504191.1	-	Dias and Sukumaran (2023)	-
*Sthenelais boa*	France	AY839587.1	-	Wiklund et al. (2005)	-
*Sthenelais boa*	Ireland	-	NIB017-21	-	Senckenberg Research Institute and Natural History Museum
*Sthenelais jeffreysii*	Norway	-	POLNB818-14	-	University of Bergen, Natural History Collections
*Sthenelais jeffreysii*	Norway	-	CRYNO077-14	-	University of Bergen, Natural History Collections
*Sthenelais limicola*	North Spain	KF808176.1		Aylagas et al. (2014)	-
*Sthenelais limicola*	Norway	-	CRYNO275-15	-	University of Bergen, Natural History Collections
*Sthenelais limicola*	North Sea	-	GEANS190-20	-	Deutsches Zentrum fuer Marine Biodiversitaetsforschung
*Sthenelais limicola*	Norway	-	POLNB1624-15	-	University of Bergen, Natural History Collections
*Sthenelais limicola*	Norway	-	PONOR163-20	-	NTNU University Museum, Department of Natural History
*Sthenelais* sp. A ZW-2021	China	OL693265.1	-	-	State Key Laboratory of Marine Environmental Science, Xiamen University
*Sthenelais* sp. A ZW-2021	China	OL964292.1	-	-	State Key Laboratory of Marine Environmental Science, Xiamen University
*Sthenelais* sp.	India	ON089671.1	-	-	Sathyabama Institute of Science and Technology
*Sthenelais tertiaglabra*	West USA	-	BBPS910-19	-	Florida Museum of Natural History
*Sthenelais verruculosa*	West USA	-	CMBIA504-11	-	Southern California Coastal Water Research Project
*Sthenelanella uniformis*	West USA	MW173680.1	-	Tilic et al. (2021)	-
*Sthenelanella uniformis*	West USA	AY894322.1	-	Struck et al. (2005)	-
*Aphrodita aculeata*	Sweden	AY839578.1	-	Wiklund et al. (2005)	-
*Mexieulepis weberi*	Belize	JN852920.1	-	Norlinder et al. (2012)	-
*Sthenelais onca* sp. nov.	Costa Rica	PX556436	BMAR008-17	Present study	Museum of Zoology, University of Costa Rica (MZUCR)
*Sthenelais onca* sp. nov.	Costa Rica	PX556435	BMAR009-17	Present study	Museum of Zoology, University of Costa Rica (MZUCR)
*Sthenelais onca* sp. nov.	Costa Rica	PX556434	BMAR014-17	Present study	Museum of Zoology, University of Costa Rica (MZUCR)
*Sthenelais onca* sp. nov.	Costa Rica	PX556433	BMAR015-17	Present study	Museum of Zoology, University of Costa Rica (MZUCR)
*Sthenelais onca* sp. nov.	Costa Rica	PX556432	BMAR6330-25	Present study	Museum of Zoology, University of Costa Rica (MZUCR)

The outgroups *Mexieulepis weberi* (Horst, 1922) (Eulepethidae) and *Aphrodita aculeata* Linnaeus, 1758 (Aphroditidae) were selected based on the phylogenetic analysis of Aphroditiformia by Norlinder et al. (2012) to appropriately root the phylogenetic tree, and the tree was edited using FigTree version 1.4.4.

The uncorrected pairwise distance of COI alignment sequences was calculated using *ape* package in RStudio with the command *dist.dna* (Paradis et al. 2004) between the Costa Rican specimens and the most similar sequences recovered in the clades of the Bayesian tree.

### Phylogenetic analysis results

**Figure 2.**
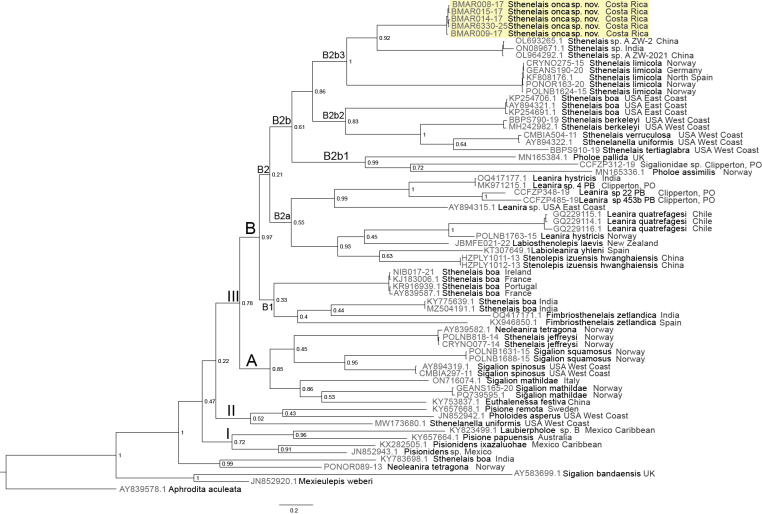
Bayesian phylogenetic tree of the family Sigalionidae based on COI sequences. Node support values are posterior probabilities. Clade labels are referred to in the text.

In the Bayesian phylogenetic analysis based on COI sequences (Fig. 2), the subfamilies Pisioninae and Pholoinae were placed in both clades, I and II. The subfamily Sthenelanellinae was also recovered in clade II as the sister group to the remaining species of the subfamily Sigalioninae (Clade III). Within this subfamily, three clades were obtained. One of these clades (Clade A, Fig. 2) contained species of *Sigalion* from the USA, Italy, and Norway; *Euthalenessa* from China; and *Sthenelais jeffreysi* M’Intosh, 1876 and *Neoleanira tetragona* (Örsted, 1845), both from Norway.

Clade B included the subclade B1 (Fig. 2), which contained *Fimbriosthenelais zetlandica* (M’Intosh, 1876) from Spain and India, two sequences of *Sthenelais boa* (Johnston, 1833) from India, and four sequences from Europe. Moreover, the support value for subclade B1 was low. The other subclade B2a (Fig. 2) included the genera *Stenolepis*, *Labioleanira*, *Labiosthenolepis*, and *Leanira*.

Subclade B2b1 contained species of *Pholoe* of the subfamily Pholoinae (Fig. 2), whereas the well-defined subclades B2b2 and B2b3 corresponded to the genus *Sthenelais* with the inclusion of one sequence of subfamily Sthenelanellinae (*Sthenelanella uniformis*). This analysis shows a separation between species of North America on the east coast with *S. boa* and the species of *Sthenelais* (*S. verruculosa*, *S. berkeleyi*, and *S. tertiaglabra*) from the west coast (subclade B2b2, Fig. 2). The focus of this study, *Sthenelais onca* sp. nov. from Costa Rica, was recovered within the well-defined subclade B2b3 and formed a grade including *Sthenelais limicola* (Ehlers, 1864) from Europe and other representative *Sthenelais* from Asia (subclade B2b3, Fig. 2). In terms of mean pairwise distance, *S. limicola* and the sequences from Asia were the most closely related to the Costa Rican specimens with divergences of 19.8% and 19.1%, respectively (see Suppl. material 2).

## Species description

### Family Sigalionidae Malmgren, 1867

**Subfamily Sigalioninae Malmgren, 1867**

**Genus *Sthenelais* Kinberg, 1856**

#### Sthenelais onca sp. nov.

Taxon classificationAnimaliaPhyllodocidaSigalionidae

26F5CA5F-C41F-5D87-B5C2-5DEBA853542E

https://zoobank.org/77E85DB9-ABF9-4F9C-9795-A2664484A7DE

Figs 3, 4, 5

##### Specimens examined.

***Holotype***: Costa Rica • Guanacaste, Naranjo Beach; 10°45'53.712"N, 85°39'27.323"W; 0 m a.s.l.; 25 Feb. 2016; J. Sibaja leg., J. C. Azofeifa leg., Y. Vega leg., G. Ampié leg.; sand, low tide, fixed in 95% ethanol; Process ID: BMAR014-17; GenBank: PX556434; MZ-UCR-387-07. ***Paratypes***: Costa Rica • 3; Guanacaste, Naranjo Beach; 10°45'53.712"N, 85°39'27.323"W; 0 m a.s.l.; 25 Feb. 2016; J. Sibaja-Cordero leg., J. C. Azofeifa leg., Y. Vega leg., G. Ampié leg.; sand, low tide, fixed in 95% ethanol; BOLD Process ID: BMAR008-17, BMAR009-17, BMAR015-17, GenBank: PX556436, PX556435, PX556433; MZ-UCR-387-01, MZ-UCR-387-02, MZ-UCR-387-08. Costa Rica • 3; Guanacaste, Naranjo Beach; 10°47'25.55"N, 85°40'31.12"W; 0 m a.s.l.; 11 May. 2024; J. Sibaja-Cordero leg., W. Miranda-García leg.; sand, low tide, fixed in 95% ethanol; BOLD Process ID: BMAR6330-25; GenBank: PX556432; MZ-UCR-2528-04. BIN: ADL0665 for all specimens.

##### Description.

**Figure 3.**
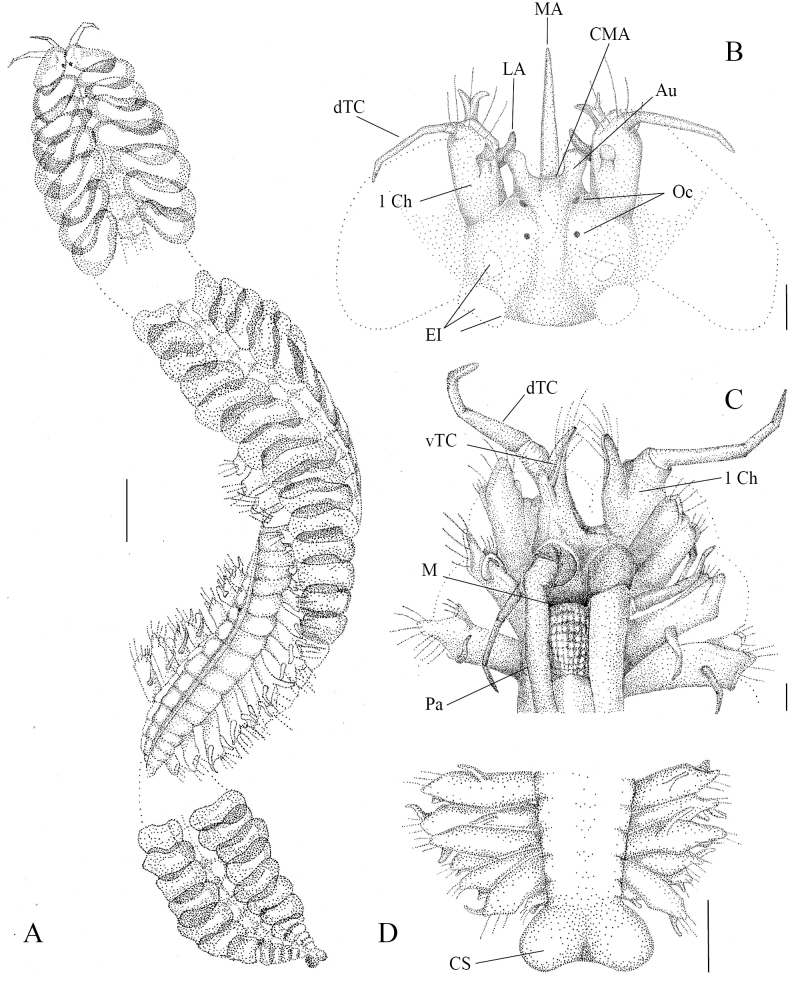
*Sthenelais onca* sp. nov., holotype (MZ-UCR 387-07). **A**. Anterior, median, and posterior body segments, dorsal view; **B**. Prostomium, dorsal view; **C**. Prostomium and peristomium, ventral view; **D**. Pygidium, dorsal view. Abbreviations: 1 Ch = first chaetiger, Au = auricle, CMA = ceratophore of median antenna, Cs = cirrus scar, dTC = dorsal tentacular cirrus, El = elytrophore, LA = lateral antenna, M = mouth, MA = median antenna, Oc = ocelli, Pa = ventral palps, vTC = ventral tentacular cirrus. Scale bars: 1 cm (**A**); 50 μm (**B**); 100 μm (**C, D**).

**Figure 4.**
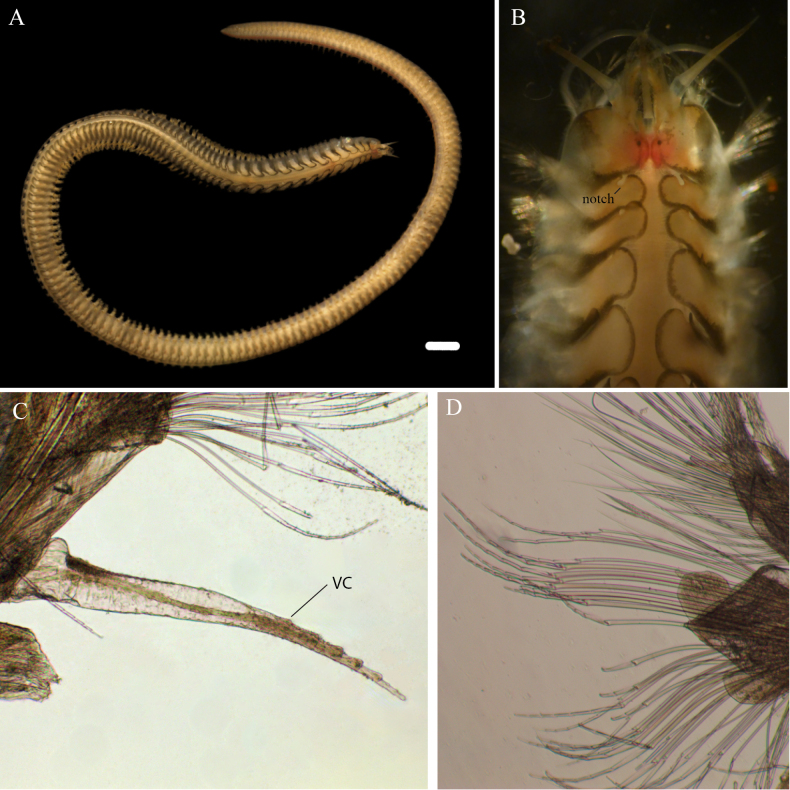
*Sthenelais onca* sp. nov. paratypes (MZ-UCR 387-02, 387-08). **A**. Paratype MZ-UCR 387-02, complete specimen; **B**. Anterior region showing live coloration and the elytral notch; **C**. Paratype MZ-UCR 387-08, detail of ventral cirrus; **D**. Paratype MZ-UCR 387-08, median chaetigers. The lower group of neurochaetae corresponds to compound falcigers with smooth shafts. Abbreviations: VC = ventral cirrus. Scale bar: 1 mm (**A**).

Holotype (MZ-UCR 387-07) complete, well preserved, 67 mm long, 3.6 mm wide, 174 chaetigers. Paratypes well preserved, (MZ-UCR 387-01, 387-08, 2528-04) complete, (MZ-UCR 387-02, 2528-04) incomplete, 22-110 mm long, 2.4-4 mm wide, 83-197 chaetigers. Left chaetigers 3, 4, and 60 extracted for characterization. Live coloration with faint orange, dorsal median line; lateral grayish longitudinal striations present. Color in alcohol yellow to whitish (Figs 3A, 4A).

***Prostomium***: Prostomium with small lateral auricles on ceratophore of median antenna (Fig. 3B). Two pairs of eyes present, one dorsal and one anterior-dorsal pair between the median antenna and first chaetiger (Fig. 3B). Palps extending ventrally to about segments 15-16 (Fig. 3C). Live coloration orange (Fig. 4B).

***Parapodia***: Biramous. Anterior parapodia closely together, slightly smaller than those of midbody segments; posterior parapodia gradually decreasing in size and becoming more closely spaced (Fig. 3A, D). First chaetiger directed anteriorly, bearing a dorsal tentacular cirrus three times larger than ventral tentacular cirrus (Fig. 3C), and a small dorsal tentacular crest (Fig. 3A).

**Figure 5.**
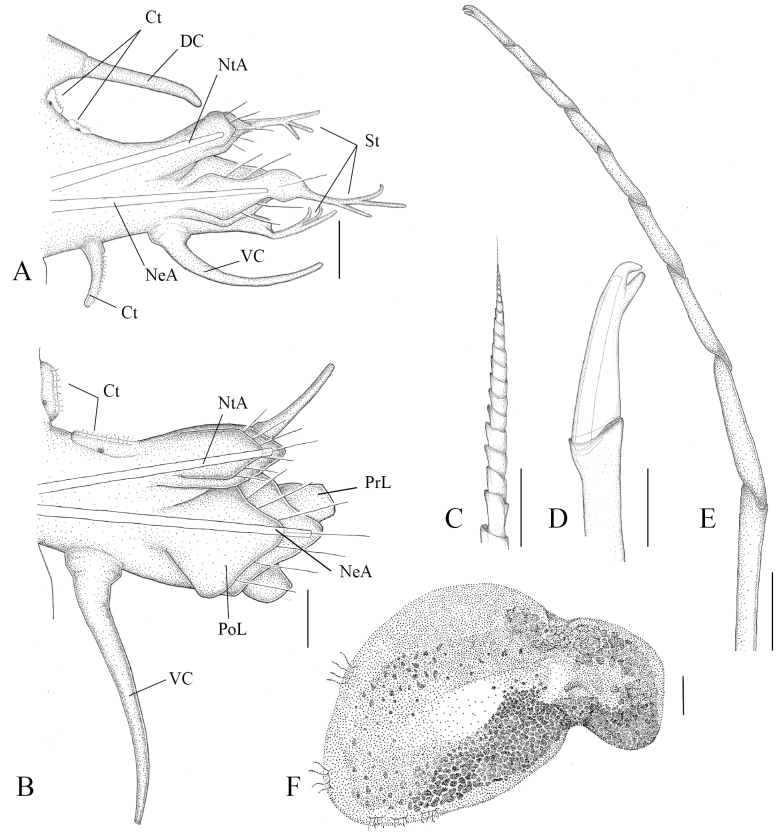
*Sthenelais onca* sp. nov., paratype (MZ-UCR 387-08). **A**. Fourth chaetiger; **B**. Median chaetiger; **C**. Supra-neuroacicular simple spinose spiniger; **D**. Sub-neuroacicular single-bladed falciger with a bifid tip; **E**. Neuropodial multiarticulate falciger with a bifid tip; **F**. Elytron from median segments. Abbreviations: Ct = ctenidium; DC = dorsal cirrus; NeA = neuroacicula; NtA = notoacicula; PoL = post-chaetal lobe; PrL = pre-chaetal lobe; St = stylodes; VC = ventral cirrus. Scale bars: 50 μm (**A, B**); 5 μm (**C, D, E**); 100 μm (**F**).

In following chaetigers, ventral cirri two times smaller than dorsal tentacular cirri, mostly smooth, with small distal protuberances (Fig. 4C). Stylodes without papillae, with digitiform extensions on notopodial bract (Fig. 5A) and on neuropodial lobes of chaetiger 4; in median and posterior segments, single stylodes present on notopodial bract and on anterodorsal bract of neuropodium.

***Chaetae***: Notochaetae serrated, with capillary unidentate tips. Neurochaetae arranged in distinct fascicles. Dorsalmost supra-neuroacicular fascicle consists of three to eight simple spinose spinigers (Fig. 5C). Remaining supra-neuroacicular fascicles with multiarticulated falcigers. Sub-neuroacicular fascicle of articulated single-bladed falcigers present (Fig. 5D), plus a more ventral fascicle of long, multiarticulate falcigers. Multiarticulate falcigers with six to eight articles (Fig. 5E). Articulate and multiarticulate falcigers bidentate. All falciger types lack serrations on shaft (Figs 4D, 5D–E).

***Elytra***: Elytra subreniform, lateral borders with a row of filiform papillae (Fig. 5F). Pale in the inner region, bordered by low, rounded, dark gray microtubercles. First pair of elytra with rounded superior margins, covering and extending beyond prostomium (Fig. 3B). Elytra absent on segments 3, 6, and 8, alternating with segment 27. Following anterior elytra with a notch (Fig. 4B) on supra-interior margin, gradually diminishing and disappearing by segment 27. Anterior elytral length decreasing posteriorly to segment 27, constant from segment 27 onwards. Cirriform ciliate branchia present lateral to elytral insertion.

***Pygidium***: Caudal cirri absent, loss cannot be ruled out (Fig. 3D).

##### Remarks.

*Sthenelais onca* sp. nov. is distinguished from other *Sthenelais* species from the region and surrounding areas by the lack of serrations on the chaetal shaft throughout the body (see Suppl. material 3); for example, *S. helenae* and *S. fusca* have multiple spinose rows on the chaetal shaft. Genetically, *S. onca* sp. nov. is most similar to *S. limicola*, and both species share smooth chaetal shaft of neurochaetae. They differ in geographic distribution and elytral morphology. *Sthenelais onca* sp. nov. has a notch on the supra-interior margin of the anterior elytra, whereas *S. limicola* has a notch on the outer margin of posterior elytra (Barnich and van Haaren 2021).

*Sthenelais onca* sp. nov. differs from congeners with smooth-shafted chaetae by its subreniform elytra with a distinct anterior notch, a pale background with a dark gray shade, and consistently bidentate compound neurochaetae. Elytral differences among these species include the absence of a notch (*S. anocula* Day, 1973) or a different position of the notch, as in *S. limicola* Ehlers, 1864 (Barnich and van Haaren 2021); differences in elytra coloration (*S. chathamensis* Knox, 1960; *S. gracilis* Verrill, 1879; *S. malayana* Horst, 1917); elytra lobulations in the margins (*S. filamentosus* Ditlevsen, 1917; *S. pectinata* Thomassin, 1970); and triangular shape of the first elytra (*S. blanchardi*). Neurochaetal differences include the absence of bidentate tips in *S. anocula*, capillary-tipped neurochaetae in *S. gracilis*, and unidentate tips in *S. filamentosus* and *S. chathamensis*.

##### Habitat.

Intertidal zone, sandy beach. Buried in sand of the surf and saturated zones. Playa Naranjo, Guanacaste, Costa Rica.

##### Etymology.

The specific epithet *onca* is a noun in apposition referring to the jaguar (*Panthera onca*), which is also found in Playa Naranjo, Costa Rica. *Sthenelais onca* sp. nov. bears elytra with a ringed pattern and a sandy and dark gray color scheme resembling the jaguar’s coat (Fig. 4A, B). This polychaete is presumed to be a predator (Rouse et al. 2022), as is the jaguar, which preys upon sea turtles that visit the beach, and other animals in Playa Naranjo (Montalvo et al. 2020).

## Discussion

For tropical America, only species of *Sthenelais* with serrated or spinose chaetal shafts throughout the parapodia have been reported. Salazar-Silva and Salazar-Vallejo (2021) stated that species from tropical America are characterized by having numerous or some rows of spines on the shafts of the neurochaetae. Nonetheless, eight *Sthenelais* species with smooth-shafted neurochaetae occur outside tropical America (see Suppl. material 3). *Sthenelais onca* sp. nov. differs from these species in one or more of the following traits: subreniform elytra with a distinct notch on the anterior margin, a pale background coloration with a dark gray shade, and compound neurochaetae that are consistently bidentate. In this way, *S. anocula* from the Atlantic coast of the USA differs by having broadly oval elytra without a notch, lacking simple bidentate neurochaetae, and lacking eyes (Day 1973). *Sthenelais gracilis* from the North Atlantic coast of the USA has white elytra and compound neurochaetae with capillary tips (Verrill 1879). In *S. filamentosus* from Iceland, the main difference is the presence of an unidentate tip in the upper long multiarticulate neurochaetae. Additionally, the anterior elytra are round, whereas the posterior ones are oblong to reniform, and the elytral margin bears two or three groups of lobations (Ditlevsen 1917).

*Sthenelais chathamensis* from New Zealand differs from *S. onca* sp. nov. in having predominantly transparent elytra, longer than wide, and upper compound neurochaetae with multiarticulate blades ending in unidentate tips (Knox 1960). With *S. pectinata* Thomassin, 1970, from the Indian Ocean, it differs mainly in that the elytra bear 6–8 longer and thicker cylindrical papillae, arranged to produce a comb-like (pectinate) appearance. The first pair of elytra is subcircular, whereas the remaining ones are reniform (Thomassin 1970). In the case of *S. malayana* from Celebes, the elytra are reniform but differ from those of *S. onca* sp. nov. in being pale red with orange tubercles (Horst 1917). Other species that exhibit differences in the elytra are *S. blanchardi*, from Chile, in which the first pair of elytra is triangular; in contrast, in *S. onca* sp. nov., the first pair has rounded superior margins. The remaining elytra in *S. blanchardi* are reniform, although an elytral notch is neither mentioned nor illustrated for this species (Kinberg 1858). Finally, *S. limicola* from the North Atlantic is discussed below because it is genetically the most closely related species to *S. onca* sp. nov., but differs in the position of the elytral notch (Barnich and van Haaren 2021).

Geographically, the distributions of *S. helenae* and *S. fusca* overlap with that of the new species (see Suppl. material 3), although important morphological distinctions are present. Both species have multiple rows of minute spines on the shafts of the neurochaetae, whereas *S. onca* sp. nov lacks serrations on the chaetal shaft throughout the parapodia. In addition, *S. fusca* has heavy, non-articulate bifid falcigers in the neuropodia, and elytra with conical microtubercles and several rows of filiform papillae (Pettibone 1971; Blake 1995), in contrast to *S. onca* sp. nov., which has light neurochaetae and a single row of filiform papillae on the lateral margin of the elytra. In *S. helenae*, as indicated by Salazar-Silva and Salazar-Vallejo (2021), the branchia are short and slightly ciliated, while in *S. onca* sp. nov. the branchia are densely ciliated.

The results of the Bayesian genetic tree suggest that *S. onca* sp. nov. (Costa Rica) is included in the major clade comprising species from Europe, Asia (subclade B2b3), and North America (subclade B2b2). North American species, such as *S. berkeleyi* and *S. tertiaglabra*, from the western coast of the USA, are characterized by falcigers with shafts bearing rows of spinelets. Additionally, *S. tertiaglabra* lacks lateral antennae, and *S. berkeleyi* has a thickly papillated ventral surface. The other species, *S. verruculosa* has a chaetal shaft ornamented with rows of delicate spines and lacks single blade falcigers in the middle and posterior neuropodia (Blake 1995). None of these characters are present in *S. onca* sp. nov. Within subclade B2b2, sequences from *S. boa* from the eastern coast of the USA are recovered, separate from European specimens. The species *S. boa* presents margins of anterior neuropodial bracts with digitiform extensions, a feature absent in *S. onca* sp. nov. Furthermore, *S. onca* sp. nov only has notochaetae with simple tips, differing from *S. boa*, which has notochaetae with simple or bidentate capillary tips (Barnich and van Haaren 2021).

The subclade B2b3 in Fig. 2 contains the species more closely related to *S. onca* sp. nov. First, it includes sequences of specimens from China and India without formal species identification in BOLD and GenBank. These Asian sequences may represent putative new species for this region. However, COI sequencing of previous reported species from the Asian region is needed to corroborate their presence, especially for species with distant type localities, such as *S. boa* reported by Izuka (1912), and *S. fusca* and *S. helenae* reported by Imajima (2011) from Japan. The same procedure is needed for species for which DNA data are currently unavailable, for example, *Sthenelais orientalis* Potts, 1910 from India, with only one spiniger in the notopodia as was described by Potts (1910), and other Indo-Pacific species of *Sthenelais* (*S. luxuriosa* Grube, 1875, *S. malayana*, or *S. nami* Gallardo, 1968) reported from South China Sea and the Philippine Sea (Salazar-Vallejo et al. 2014). Second, within clade B2b3, *S. limicola* has irregular extensions in the outer margin of the anterior elytra and a notch in the outer margins of posterior elytra (Barnich and van Haaren 2021). While in *S. onca* sp. nov., a notch is present on the supra-interior margin of the elytra from the second pair to segment 27. Additionally, compound falcigers in the middle neurochaetae of *S. limicola* may have a short, uni- or bi-articulated blade (Barnich and van Haaren 2021), whereas bi-articulated blades are not present in *S. onca* sp. nov.

Due to the vast geographic distance between the distribution of type localities of several *Sthenelais* species and *S. onca* sp. nov. from the Pacific coast of Costa Rica, and the morphological and genetic differences from other species present in the Eastern Tropical Pacific, *Sthenelais onca* is herein recognized as a new species. An updated identification key to the species of *Sthenelais* in tropical America is also provided.

### Key to the species of *Sthenelais* Kinberg, 1856

Key to the species of *Sthenelais* Kinberg, 1856 of tropical America, modified from Salazar-Silva and Salazar-Vallejo (2021), including *Sthenelais onca* sp. nov. Abbreviations: **B** = Baja California; **Br** = Brazil; **C** = Caribbean Sea; **ETP** = Eastern Tropical Pacific; **G** = Gulf of Mexico; **Q** = questionable reports.

**Table:** 

1	Shaft of the neurochaetae of intermediate segments smooth, anterior elytra bearing a notch on the supra-interior margin	***Sthenelais onca* sp. n (ETP)**
–	Shaft of the neurochaetae of intermediate segments with rows of spines, anterior elytra bearing a notch in a different position	**2**
2(1)	Shaft of the neurochaetae of median segments with numerous rows of spines	**3**
–	Shaft of the neurochaetae of median segments with few rows of spines	**6**
3(2)	Ventral surface with papillae	***S. berkeleyi* Pettibone, 1971 (ETP)**
–	Ventral surface without papillae	**4**
4(3)	Neurochaetae articulated with 10 to 15 articles, one or two falcigers	***S. tertiaglabra* Moore, 1910 (B, ETP)**
–	Neurochaetae articulated with fewer than 10 articles, numerous falcigers	**5**
5(4)	Elytra with conical microtubercles uniformly distributed; marginal papillae along the entire outer edge; nuchal organs concealed by segment two	***S. articulata* (Kinberg, 1856) (Br; C, Q)**
–	Elytra with scattered microtubercles, marginal papillae sparse along the outer edge; nuchal organs not concealed	***S. setosa* Bush in: Verrill, 1900 (C)**
6(2)	Neurochaetae with bifid tips and some with entire tips; posterior elytra with 3–4 sclerotized conical tubercles on the posterior margin	***S. neoleanirae* Hartman, 1939 (ETP)**
–	Neurochaetae only bifid; posterior elytra with tubercles in a different arrangement	**7**
7(6)	Median or posterior elytra with a single row of papillae along the lateral margin, microtubercles predominantly globular	***S. verruculosa* Johnson, 1897 (ETP)**
–	Median or posterior elytra with several rows of papillae along the lateral margin, microtubercles of another form	**8**
8(7)	Lateral margin of elytra with papillae arranged in up to six submarginal rows; short, non-articulated falcigers more numerous than articulated ones; simple neurochaetae absent	***S. fusca* Johnson, 1897 (ETP, B)**
–	Lateral margin of elytra with papillae in two submarginal rows; non-articulated falcigers in a different arrangement; simple neurochaetae present	**9**
9(8)	Parapodia with short, slightly ciliated branchiae; stylodes scattered on neuropodia	***S. helenae* Kinberg, 1856 (ETP; C, Q)**
–	Parapodia with long, ciliated branchiae; stylodes numerous on neuropodia	***S. maculata* Hartman, 1939 (ETP; B, Q; C, Q)**

## Supplementary Material

XML Treatment for Sthenelais onca

## Additional information

### Artificial Intelligence (AI) use

The authors accept full responsibility for the content of the manuscript, including the disclosure of any use of AI.

No AI tools were used in the preparation of this manuscript.

### Author ORCIDs

J.A. Sibaja-Cordero https://orcid.org/0000-0001-5323-356X

W. Miranda-García https://orcid.org/0009-0009-0305-6724

### Data availability

All of the data that support the findings of this study are available in the main text or Supplementary Information.

## Supplementary materials

Supplementary material 1 *Sthenelais* nexus Data type nex Explanation note A COI sequence analysis for MrBayes of species of *Sthenelais*. https://binary.pensoft.net/file/1607418This dataset is made available under the Open Database License (http://opendatacommons.org/licenses/odbl/1.0/). The Open Database License (ODbL) is a license agreement intended to allow users to freely share, modify, and use this Dataset while maintaining this same freedom for others, provided that the original source and author(s) are credited. Jeffrey A. Sibaja-Cordero

Supplementary material 2 Genetic distance Data type xlsx Explanation note Genetic distance based on uncorrected pairwise distance between species within Clade B2b. Mean distance among *Sthenelais onca* sp. nov. and other *Sthenelais* specimens in bold. https://binary.pensoft.net/file/1607419This dataset is made available under the Open Database License (http://opendatacommons.org/licenses/odbl/1.0/). The Open Database License (ODbL) is a license agreement intended to allow users to freely share, modify, and use this Dataset while maintaining this same freedom for others, provided that the original source and author(s) are credited. Jeffrey A. Sibaja-Cordero

Supplementary material 3 *Sthenelais* species distribution and neurosetae characteristics Data type xlsx Explanation note Distribution of species of *Sthenelais*, and neurosetae characteristics with references to original species descriptions. https://binary.pensoft.net/file/1607420This dataset is made available under the Open Database License (http://opendatacommons.org/licenses/odbl/1.0/). The Open Database License (ODbL) is a license agreement intended to allow users to freely share, modify, and use this Dataset while maintaining this same freedom for others, provided that the original source and author(s) are credited. Jeffrey A. Sibaja-Cordero, Waiomi Miranda-García
